# Anatomic Image-Based Classification of Lumbar Intervertebral Disc Pathologies

**DOI:** 10.7759/cureus.16861

**Published:** 2021-08-03

**Authors:** Said G Osman

**Affiliations:** 1 Surgery, Frederick Memorial Hospital, Frederick, Maryland, USA

**Keywords:** anatomic, image-based, lumbar, disc, topography, morphology, spinal endoscopy, trans-iliac, transforaminal, interlaminar

## Abstract

Introduction

Several minimally invasive spine approaches and techniques have been developed in recent years. While the disease processes affecting the spinal motion segment have remained largely the same, the emerging technologies have changed treatment options radically and not necessarily in an organized fashion. The current diagnostic techniques, also evolving, have helped us appreciate the disease's pathoanatomy in minute details. A comprehensive classification method accounting for all anatomical variations in the disc disease, tailored to treatment options, is necessary. Such a classification will allow the surgeon to choose an appropriate surgical option in a consistent fashion. We feel that our classification system will help the spine surgeon make that important decision consistently, with minimal risk of leaving behind a significant lesion or disrupting an otherwise normal structure of the spinal motion segment. Furthermore, we feel such a comprehensive classification will help surgeons and other caregivers to standardize treatment approaches to the various presentations of disc disease, and apply the evolving technology in an organized fashion.

Purpose

To develop a comprehensive, treatment-orientated classification of the lumbar disc disease.

Materials and Methods

The literature was reviewed for the classification of disc disease. The morphology of the disc disease, the topography of the disc lesion, and the symptom-complex produced by the disc lesion are identified and graded. The features so identified and graded are placed in a matrix. The combinations of the anatomical features and symptoms are then computed as shown in the matrix. The MRI database held in the office was studied to determine the most frequent combinations of the disc disease and symptom complex.

Results

A total of 494 combinations were identified, but most have no clinical relevance. The retrospective study of the clinical data and MRI studies of 93 patients (50 male and 43 female) revealed the most affected motion-segment was L5-S1 (male = 19.3%, and female = 23.8%). The most common patho-anatomy is a globally bulging disc (T3L1), representing 37.6% of the total. The second most common combination is a degenerated disc with central, intra-annular tear T4L1), representing 20.4% of the total. At 11.8%, globally bulging with severe axial pain and moderate radicular pain represented the most common patho-anatomic/clinical classification (T3L1B4R2). The most frequent top 10 patho-anatomic/clinical classifications represented 15.5% of the total.

Conclusion

In light of the multiple surgical options for excision of the herniated lumbar disc, including the conventional and minimally invasive, and the fact that the imaging technology allows spine surgeons to see in great detail, the disease status of each of the components of the spinal motion segment, it is imperative to develop comprehensive classification systems which take account of the unique characteristics of the disease entity and guide treatment strategies. The classification system presented here is fairly complex, but the software technology will be utilized for the classification system along with the most appropriate treatment approach.

## Introduction

This article was previously presented as an abstract in the May/June 2015 Pain Physician Journal.

Recently, Osman, et al. presented a novel comprehensive anatomic, image-based classification of lumbar spinal motion segment degeneration [1]. That classification grades the patho-anatomy of the intervertebral disc, facet joint, ligamentum flavum, and alignment of the spinal motion segment, presenting the combinations of the four structural abnormalities as a code. Along with the patient’s complete clinical data, the classification is then used to determine appropriate surgical options in a hierarchical manner - with the least invasive and most effective option being the first offered. Based on that classification system, the disc may be the only abnormal structure, and the symptoms may be entirely or mostly related to the disc abnormality. This treatment-based classification of the lumbar intervertebral disc pathology is used in conjunction with the motion-segment classification for comprehensive management of the motion-segment disease. Hitherto, diseases of the lumbar intervertebral disc have been classified according to various attributes of the condition. Spengler et al. [2] classified disc pathology according to its morphology - protrusion (contained within the annulus), extrusion (annulus torn but herniation in continuity with disc interior), and sequestrated.

Disc rupture may be classified according to its topographic location - central, lateral recess, foraminal and extra-foraminal. Disc herniations have also been classified according to the timing of the surgical intervention as this has prognostic significance [3-5]. Disc herniation may occur early in the degenerative cascade or late in the degeneration of the disc. The classifications currently available convey valuable information about the nature of herniation; however, they are all stand-alone classifications that fail to take into account the other players in the spinal motion segment. Until recently, the approach to a herniated lumbar disc or spinal stenosis has been posteriorly, through open laminotomy or laminectomy, hence, there was no need for complex classification of the herniated disc for the purpose of determining the surgical approach. However, over the last couple of decades, a number of minimally invasive treatment options for lumbar disc herniations have emerged [6-9]. These include, but are not limited to, the percutaneous endoscopic transforaminal approach, the percutaneous endoscopic interlaminar approach, and the mini-open interlaminar approach. Osman et al., in a cadaver study, described a detailed endoscopic transforaminal approach to all levels from the upper thoracic spine to the sacrum [10]. In another cadaver study, the authors also demonstrated the superiority of the transforaminal endoscopic approach as compared to laminectomy and partial facetectomy, both in terms of better foraminal decompression, maintenance of motion-segment stability, and minimalization of collateral structural damage [11]. Unlike the traditional laminectomy and laminotomy where the pre-incision concerns are mainly the identification of the level and side of the lesion, with the new minimally invasive techniques many additional considerations are critical. These include the topographical location of disc lesion, its morphology, migration of free fragment herniation, and the direction of the migration. Unfortunately, as these very useful, tissue-preserving techniques emerge, there is no universal system of classification to determine which approach is most suitable for a given disc pathological configuration. This state of affairs creates confusion in the minds of those who want to learn these techniques and make progress in the harnessing of the new technologies for the benefit of their patients. While all the new developments are exciting, it is important to appreciate that the new technologies may not be able to solve some of the problems we encounter in disc surgery. It is the aim of this classification to detail the anatomical as well as the sign and symptoms of the patient to give recommendations for the most appropriate surgical approaches in a hierarchical manner. 

## Materials and methods

The morphologic and topographic aspects of the intervertebral disc and the clinical manifestations of the disc pathology are graded as listed in Table 1.

**Table 1:** Anatomic Classification of Disc Pathology and Grading of Clinical Findings

Type of disc ruptures	Location of disc rupture	Radicular symptom/sign	Back pain (VAS score)
T1 = Acute Intra-annular	L1 = Central herniation	R0 = None	B0 = 0
T2 = Acute Extra-annular	L2 = Paracentral - Pre-dural	R1 = Mild paresthesia.	B1 = 1-2/10
T3 = Degenerated, globally bulging	L3 = Paracentral - Axillary	R2 = Moderate pain, 4/5 muscle power.	B2 = 3-6/10
T4 = Degenerate Intra-annular	L4 = Paracentral - Pre-Radicular	R3 = Severe pain, 3/5 muscle power	B3 = 7-9/10
T5 = Degenerate, Extra-annular	L5 = Intra- extra foraminal	R4 = Numb, = 0-2/5 muscle power	B4 = 10/ 10

The gradings of the patho-anatomy (morphology and topography), radicular symptoms/signs, and axial pain are placed in a matrix and the combinations are recorded as shown in Table 2.

**Table 2:** Matrix of Pathoanatomy and Clinical Gradings

	Acute Intra-annular- (T_1_)	Acute-Extra-annular - (T_2_)	Global bulge (T_3_)	Deg Intra-annular (T_4_)	Deg-Extra-annular (T_5_)	
Central herniation (L_1_)	L_1_T_1_B_0_R_0_	L_1_T_2_B_0_R_I_	L_1_T_3_B_0_R_2_	L_1_T_4_B_0_R_3_	L_1_T_5_B_0_R_4_	Back pain – (B_0_)
PC-Pre-dural (L_2_)	L_2_T_1_B_1_R_0_	L_2_T_2_B_1_R_1_	L_2_T_3_B_2_R_2_	L_2_T_4_B_1_R_3_	L_2_T_5_B_1_R_4_	Back pain – (B_1_)
PC-Axillary (L_3_)	L_3_T_1_B_2_R_0_	L_3_T_2_B_2_R_1_	L_3_T_3_B_2_R_2_	L_3_T_4_B_2_R_3_	L_3_T_5_B_2_R_4_	Back pain – (B_2_)
PC-Pre-Radicular –(L_4_)	L_4_T_1_B_3_R_0_	L_4_T_2_B_3_R_1_	L_4_T_3_B_3_R_2_	L_4_T_4_B_3_R_3_	L_4_T_5_B_3_R_4_	Back pain – (B_3_)
Intra- extra foraminal (L_5_)	L_5_T_1_B_4_R_0_	L_5_T_2_B_4_R_1_	L_5_T_3_B_4_R_2_	L_5_T_4_B_4_R_3_	L_5_T_5_B_4_R_4_	Back pain – (B_4_)
	Radcular – (R _0_)	Radicular – (R_1_)	Radicular- (R2)	Radicular – (R_3_)	Radicular – CES (R_4_)	

Based on the patho-anatomic identifications, the MRI images and drawings of the lumbar discs are presented in Figures 1 to 10.

**Figure 1:**
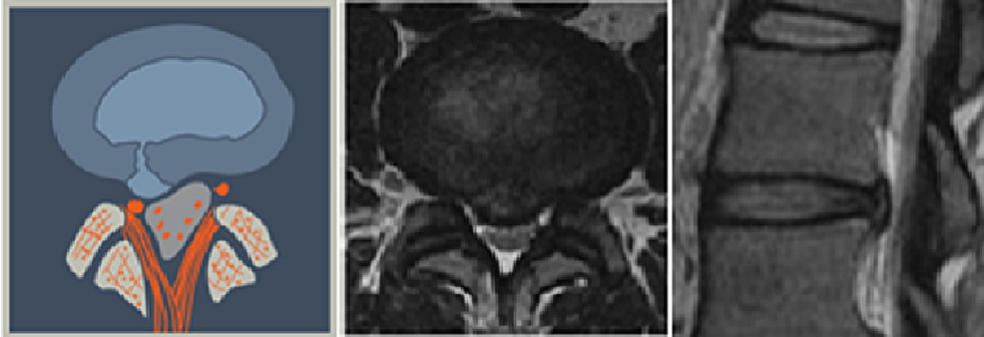
Acute Intra-Annular Tear (T1) This is the rupture of a normal disc where the protruding nuclear material is contained within the annular wall.

Figure 1 shows intra-annular tear, with the nuclear protrusion being contained within the outer layers of the annular wall.

 

**Figure 2:**
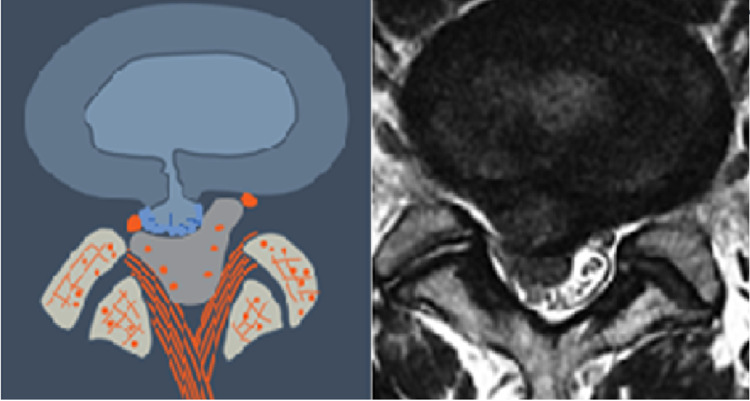
Acute Extra-Annular Tear (T2) This is rupture of a normal disc where the nuclear material extrudes through the annulus and may be a free fragment.

 

**Figure 3:**
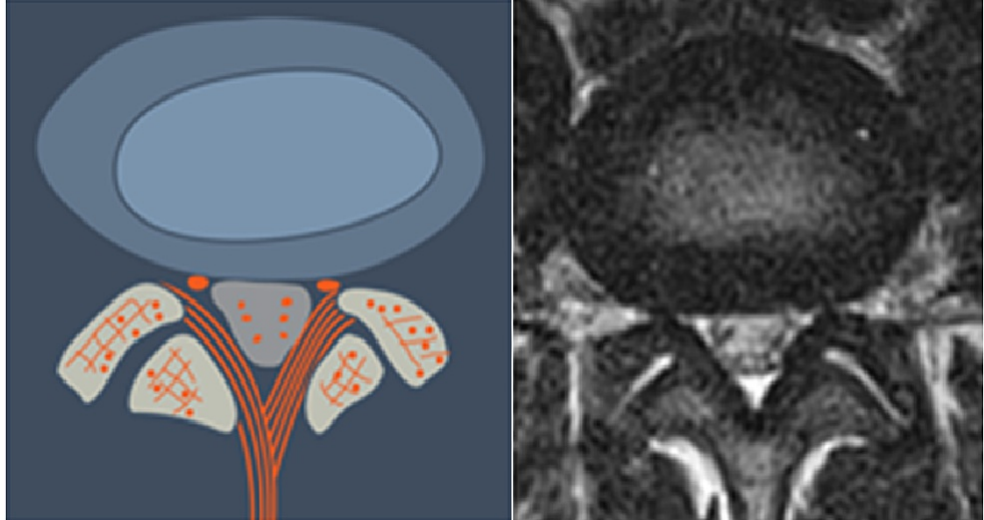
Global Disc Bulge (T3) This is a degenerated disc which bulges globally. The disc height is usually reduced. The nuclear materal is contained within the bulging annulus.

**Figure 4:**
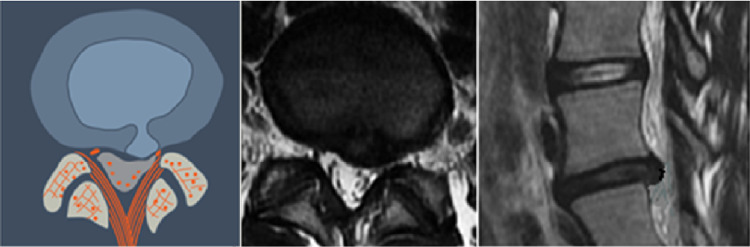
Intra-Annular Herniation (T4) This is a degenerated disc with weakend annular where the herniated nuclear fragment is contained within the annular wall.

**Figure 5:**
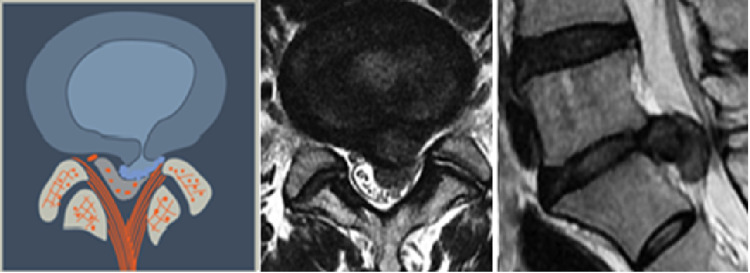
Extra-Annular Herniation, Degenerated Disc (T5) This is a degenerated disc which weakened annular wall. The nuclear material has extruded through the annulus and may migrate caudally or cranially.

**Figure 6:**
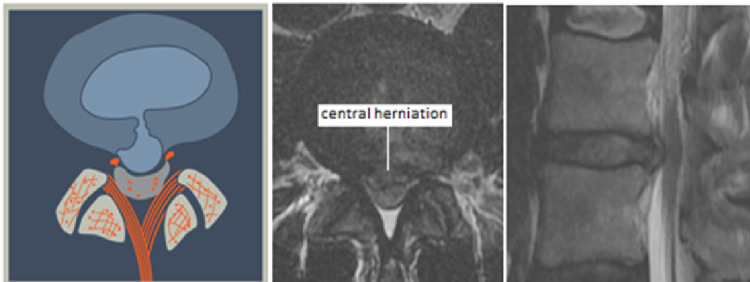
Central Herniation (L1) This may be a normal or degenerated disc which ruptures into the center of the spinal canal, directly ventral to the posterior longitudinal ligament. The herniated nuclear material may be contained with the annular wall (intra-annular) or extruded though the annulus (extra-annular).

**Figure 7:**
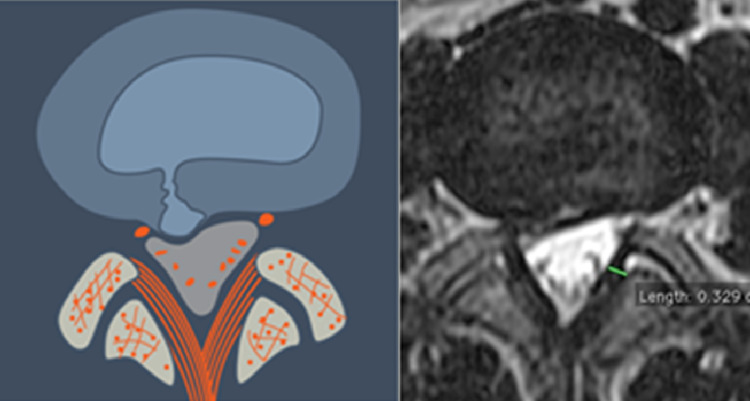
Paracentral, Predural Herniation (L2) The herniation is paracentral and may be on the right or the left side of the midline. Topographically it is found ventral to the dura and may extend to the midline.

**Figure 8:**
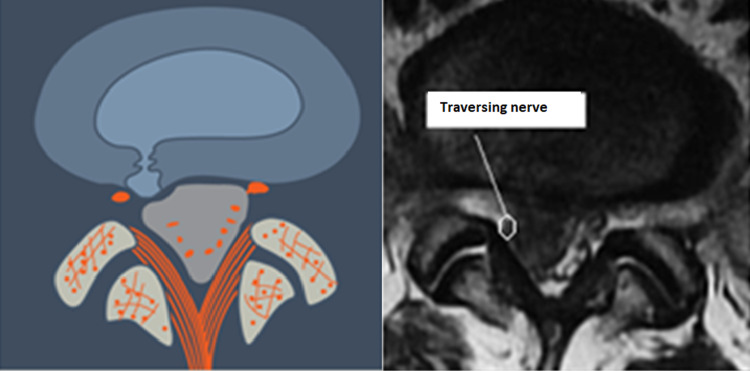
Paracentral, Axillary (L3) The herniation is paracentral and is lodged between the dura, medially, and the traversing nerve root laterally. The herniated material may be intra-annular or extra-annular (i.e. extruded).

**Figure 9:**
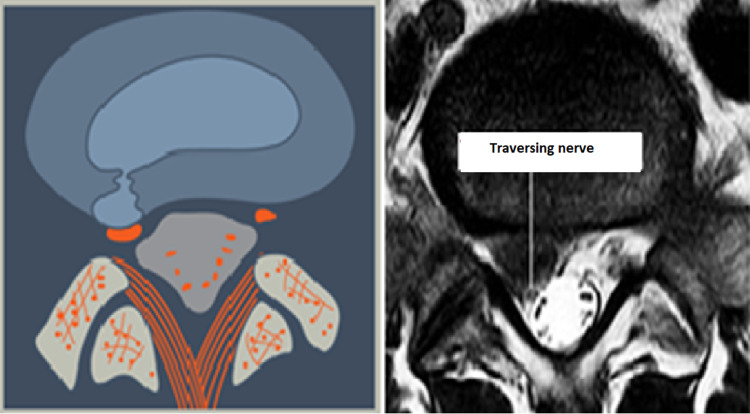
Paracentral, Pre-Radicular Herniation (L4) The herniation is paracentral and situated ventral to the  traversing nerve. The nerve is usually draped around the herniated material making it vulnerable to injury when approached posteriorly.

**Figure 10:**
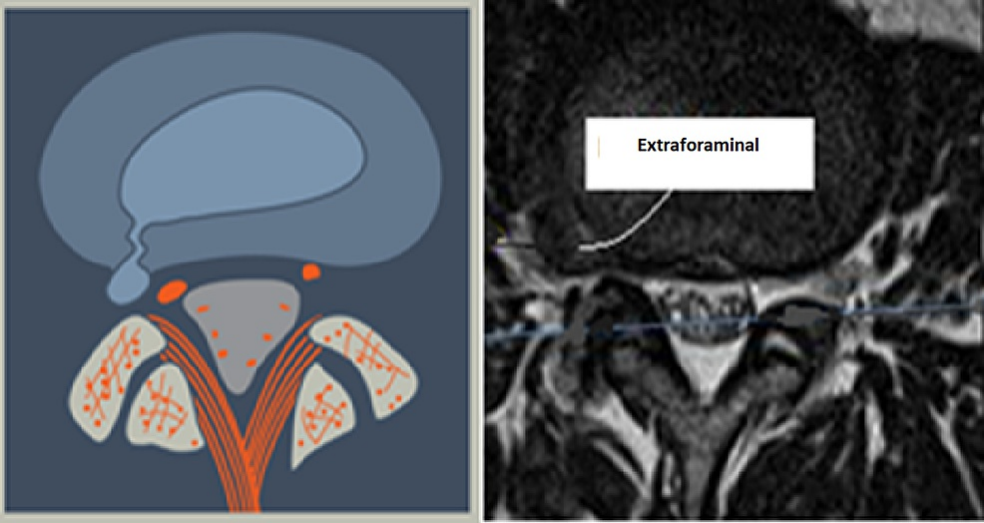
Intra-/Extra-Foraminal Herniation (L5) The herniation may be in the foraminal or extraforaminal location. This may be a normal disc or degenerated disc which ruptured, and the nuclear material may be intra- or extra-annular. The hernia usually is very close to the exiting nerve root. especially if it is extraforaminal.

## Results

A total of 494 combinations were identified using the matrix in Table 2. Most of the combinations have no clinical relevance, hence, a retrospective study of the office database was carried out to determine the prevalence of the various patho-anatomic combinations. The medical records and lumbar MRI films of 93 patients were studied (50 male and 43 female). The age range was 28-79 years. The most affected motion segment was L5-S1, both in male and female populations - 19.3% and 23.8%, respectively (Table 3). 

**Table 3:** Prevalence of Disc Lumbar Disc Disease Based on the Motion-Segment Levels.

Level	Sex				Total	
	M		F			
	#	%	#	%	#	%
L5-S1	17	19.3	21	23.9	38	43.2
L4-5	16	18.2	16	18.2	32	36.4
L3-4	6	6.8	4	4.5	10	11.4
L2-3	4	4.5	1	1.1	5	5.7
L1-2	1	1.1	2	2.3	3	3.4
Total	44	50	44	50	88	100

The most common disc patho-anatomic combination (i.e., morphology and topography) degenerates and globally bulging disc (T3L1), representing 37.6% of the total (Table 4). The next most common combination of the patho-anatomy is the central intra-annular herniation of a degenerated disc, representing 20.4% (T4L1).

**Table 4:** Common combinations of the patho-anatomic lumbar disc morphology and topography.

Level	Sex				Total	
	M		F			
	#	%	#	%	#	%
T3L1	18	19.4	17	18.3	35	37.6
T4L1	10	10.8	9	9.7	19	20.4
T4L4	7	7.5	5	5.4	12	12.9
T5L4	5	5.4	3	3.2	8	8.6
T4L5	3	3.2	3	3.2	6	6.5
T4L2	4	4.3	1	1.1	5	5.4
T5L2	0	0.0	4	4.3	4	4.3
T5L3	1	1.1	1	1.1	2	2.2
T5L1	1	1.1	0	0.0	1	1.1
T3L4	1	1.1	0	0.0	1	1.1
Total	50	53.8	43	46.2	93	100

Table 5 shows the most common gradings of the lumbar disc patho-anatomy and clinical findings. At 11.8%, globally bulging, degenerated disc presenting with severe lumbar pain and moderate radicular pain (T3L1B4R2) is the most common classification. The ten most frequent classifications, representing 15.5% of the total patho-anatomic and clinical grading, included degenerated and globally bulging discs with or without intranuclear, central herniation.

**Table 5:** Prevalence of patho-anatomic and clinical features classification.

Path/Clin Comb	Sex				Total	
	M		F			
	#	%	#	%	#	%
T3L1B4R2	7	7.5	4	4.3	11	11.8
T3L1B3R2	5	5.4	2	2.2	7	7.5
T4L1B3R2	2	2.2	5	5.4	7	7.5
T4L4B3R2	3	3.2	4	4.3	7	7.5
T5L4B2R3	3	3.2	2	2.2	5	5.4
T4L1B4R2	2	2.2	2	2.2	4	4.3
T3L1B4R3	2	2.2	1	1.1	3	3.2
T4L5B2R2	1	1.1	2	2.2	3	3.2
T3L1B2R1	0	0.0	2	2.2	2	2.2
T3L1B2R3	1	1.1	1	1.1	2	2.2

## Discussion

After comprehensive non-operative treatment, including activity modifications, pain medication, rehabilitation measures, and therapeutic injections have been utilized, persistent symptoms may require surgical intervention. The spectrum of procedures practiced today for lumbar discectomy includes percutaneous discectomy [12-14], endoscopic transforaminal discectomy [15], interlaminar endoscopic approaches [16,17], laminotomy, laminectomy [18-20], and variable degrees of facetectomy. Posterior procedures including laminotomy and laminectomy involve some degree of paraspinal muscle mobilization, excision of bony structures, and traversing the spinal canal to gain access to the pathologic disc, ventral to the dura. The consequences of such interventions may include damage to the paraspinal musculature, compromise spinal stability, and creation of epidural scar which may render subsequent surgical intervention difficult. This anatomic treatment-based disc disease classification system presents image-based morphologic and topographic grading combined with the patient's symptom-complex, and helps guide surgical approach for predominantly radicular deficits while incurring the least amount of disruption of normal structures. Based on the morphology, topography, and migration of a free fragment herniation, and other considerations, the surgeon may choose a transforaminal, interlaminar, open, or endoscopic approach. The comprehensive spinal-motion segment classification, recently described by the author [1] should be utilized to determine the appropriate surgical option if other structures of the spinal-motion segment are implicated in the symptom complex.

With such a comprehensive anatomic treatment-based classification it is hoped that the diverse technological advancement [6, 7, 11, 13] in the surgical treatment of the lumbar spine will be applied in a rational manner, and that spine professionals can compare the relative effectiveness of the various surgical options for the different structural characteristics of the disc lesion in precise anatomic fashion as opposed to the vague characterization of the patho-anatomy practiced currently.

Often, surgeons see patients who present with radicular pain but minimal or no back pain. A review of the imaging studies, however, may reveal degeneration of the facet joints associated with foraminal stenosis and some mal-alignment of the motion segment. These patients are likely to be middle-aged or elderly. In spite of the findings on the images, limiting surgical intervention to relieve the radicular pain is often sufficient. Hence, it is critical that the pain generator is precisely defined utilizing an image-based, anatomic classification of spinal motion segments [1]. The classification grades the degree of abnormality of each component of the spinal motion segment. With pre-operative diagnostic/therapeutic injections the pain generator can be accurately determined and targeted with the least invasive surgical technique if needed. 

## Conclusions

As the understanding of the patho-anatomy and pain generators improve, as the risks and benefits of different surgical approaches become appreciated, and as the cost-benefit analysis of each approach become appreciated, with the evolution of the least invasive spine technologies, this anatomic classification along with anatomic motion-segment classification will make it possible to make appropriate choices of surgical approaches. Furthermore, such anatomic classification systems will make the comparisons of different surgical approaches more accurate and, thus, help the surgeon make appropriate surgical approach decision.
